# Supplementary figures and images for: The effects of simple graphical and mental visualization of lung sounds in teaching lung auscultation during clinical clerkship: A preliminary study
Source: PLoS One. 2023 Mar 17;18(3):e0282337. doi: 10.1371/journal.pone.0282337 (PMC10022769; doi:10.1371/journal.pone.0282337)

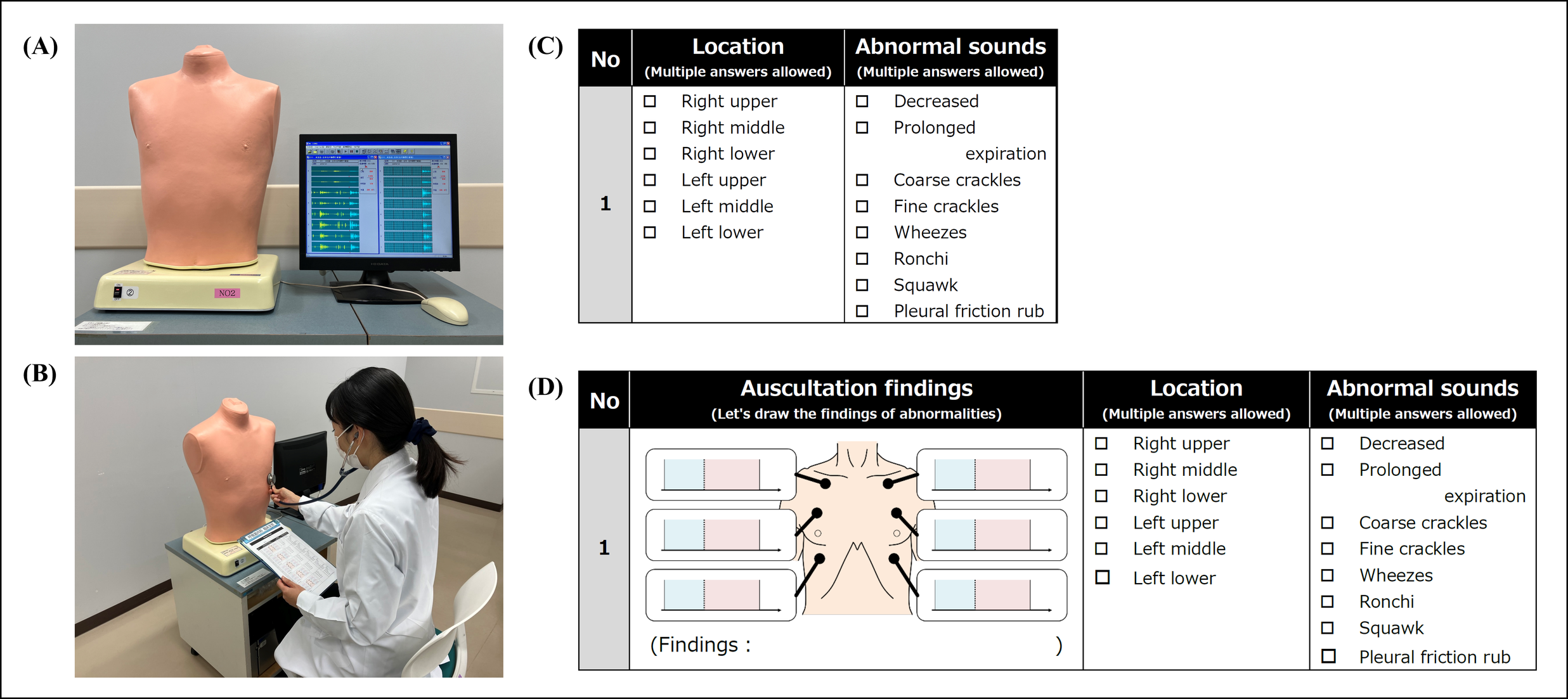

Supplement: S1 Fig — The simulator (A. Mr. Lung®, Kyoto Kagaku Co. Ltd., Kyoto, Japan) was used for lectures before CC and during CC in Respiratory medicine, the pre- and pos- test (B). Answer sheet for lung sound auscultation test. A multiple-choice form (C) was used as the pre-test for the control and visualization group and the post-test for the control group. A multiple-choice form that combined a space in which to draw a figure where there was an abnormality (D) was used as the post-test for the visualization group. CC, clinical clerkship. (TIF) [file pone.0282337.s001.tif]

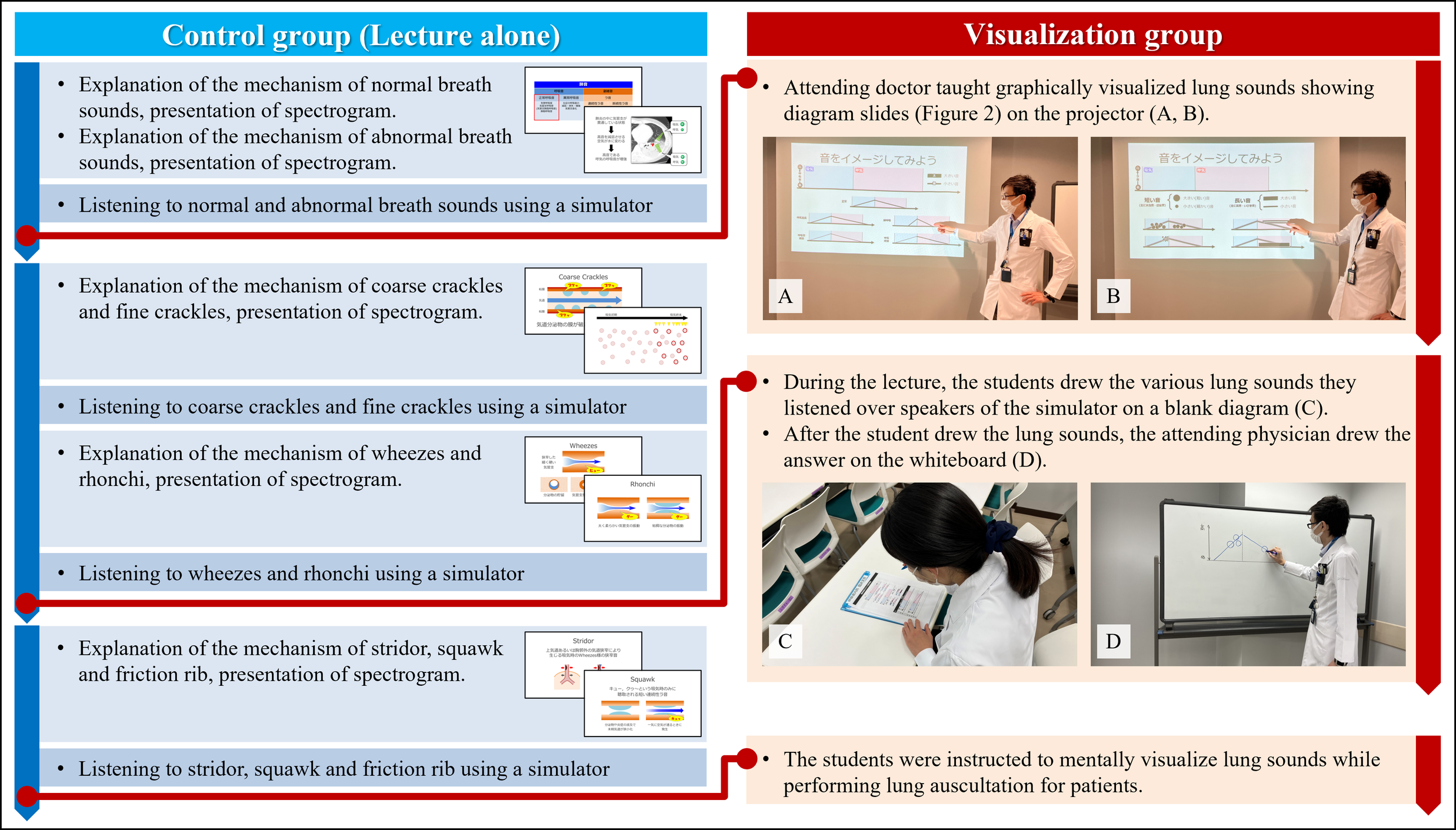

Supplement: S2 Fig — (TIF) [file pone.0282337.s002.tif]

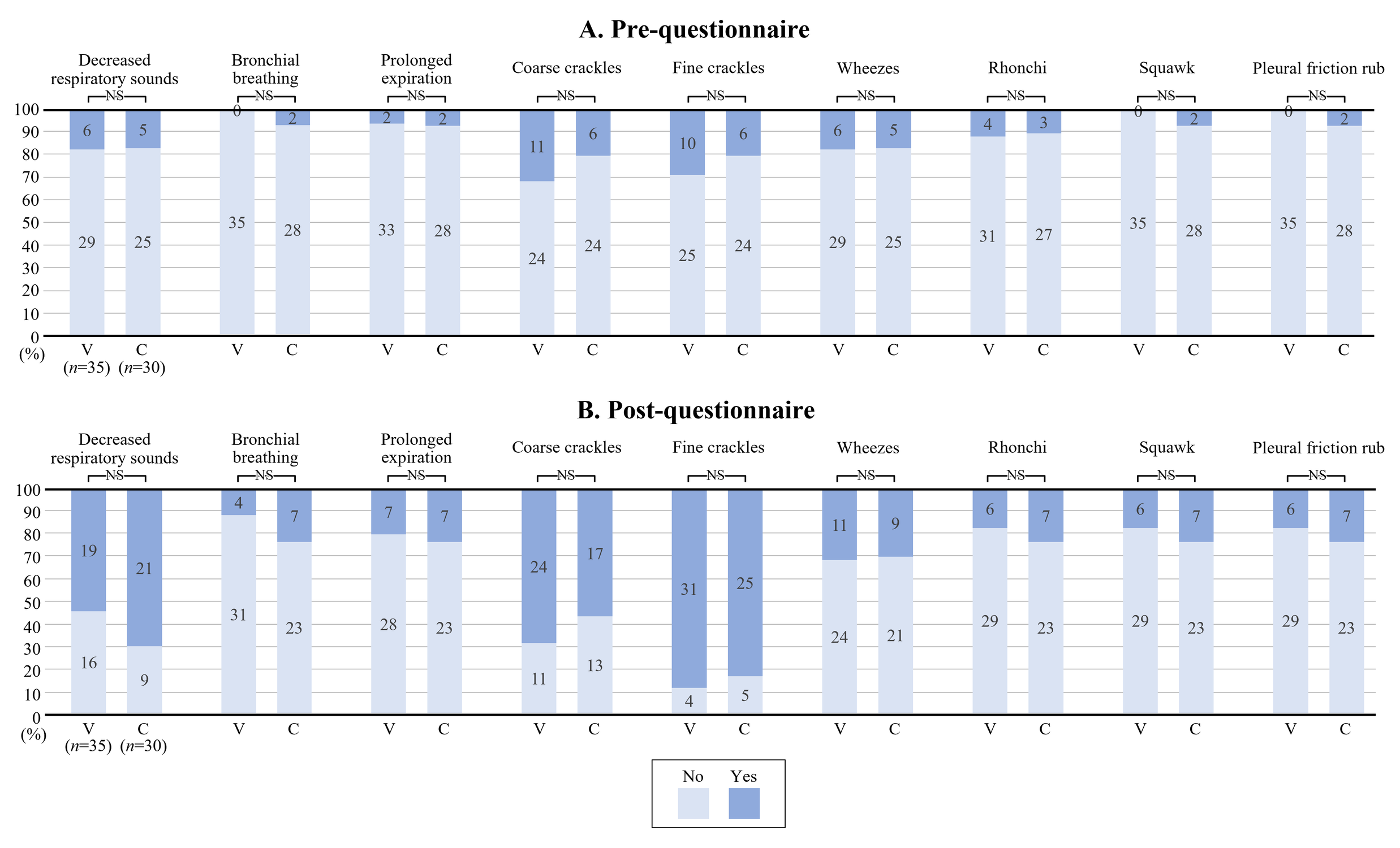

Supplement: S3 Fig — C, control group; NS, not significant; V, visualization group. (TIF) [file pone.0282337.s003.tif]

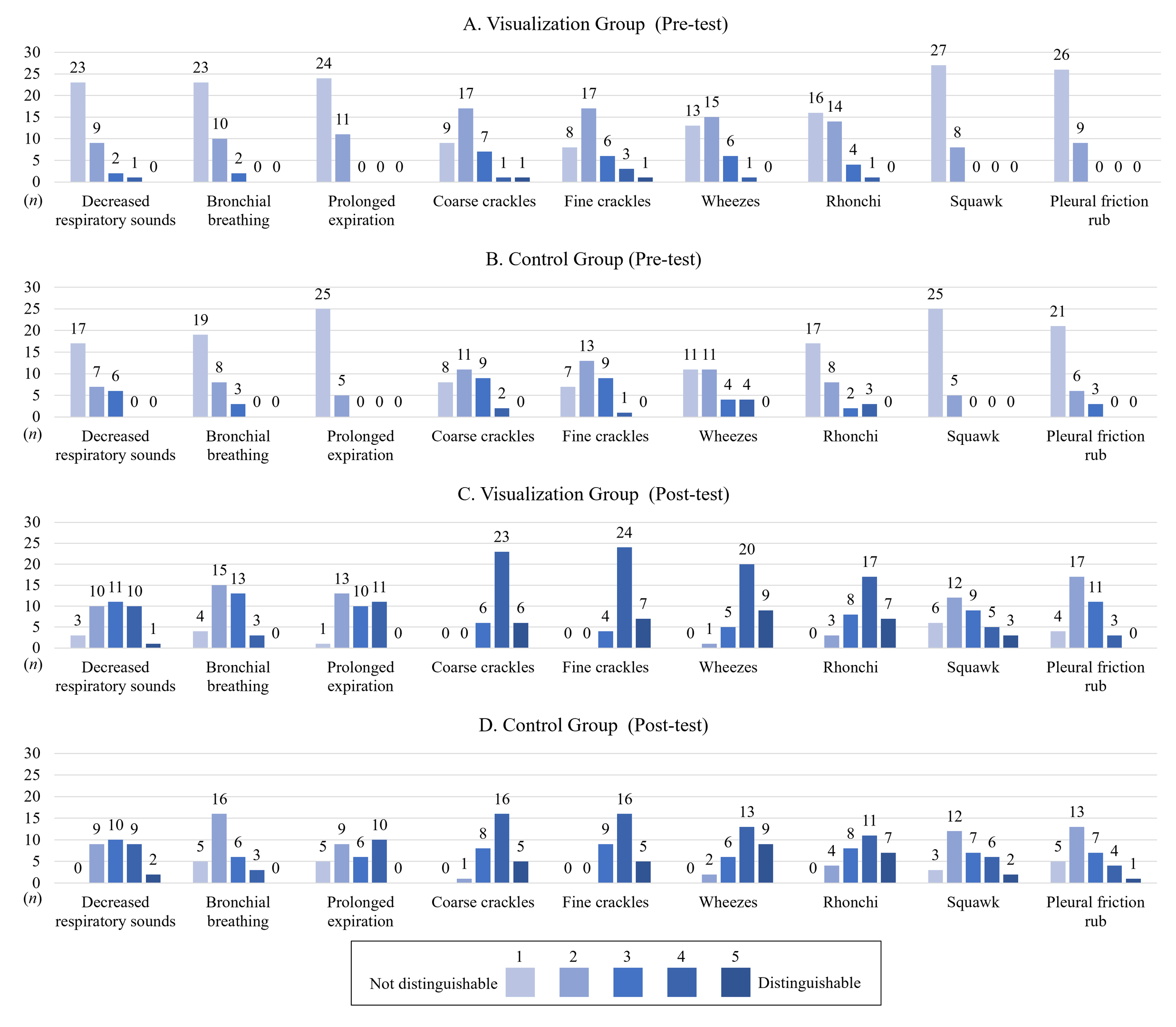

Supplement: S4 Fig — (TIF) [file pone.0282337.s004.tif]

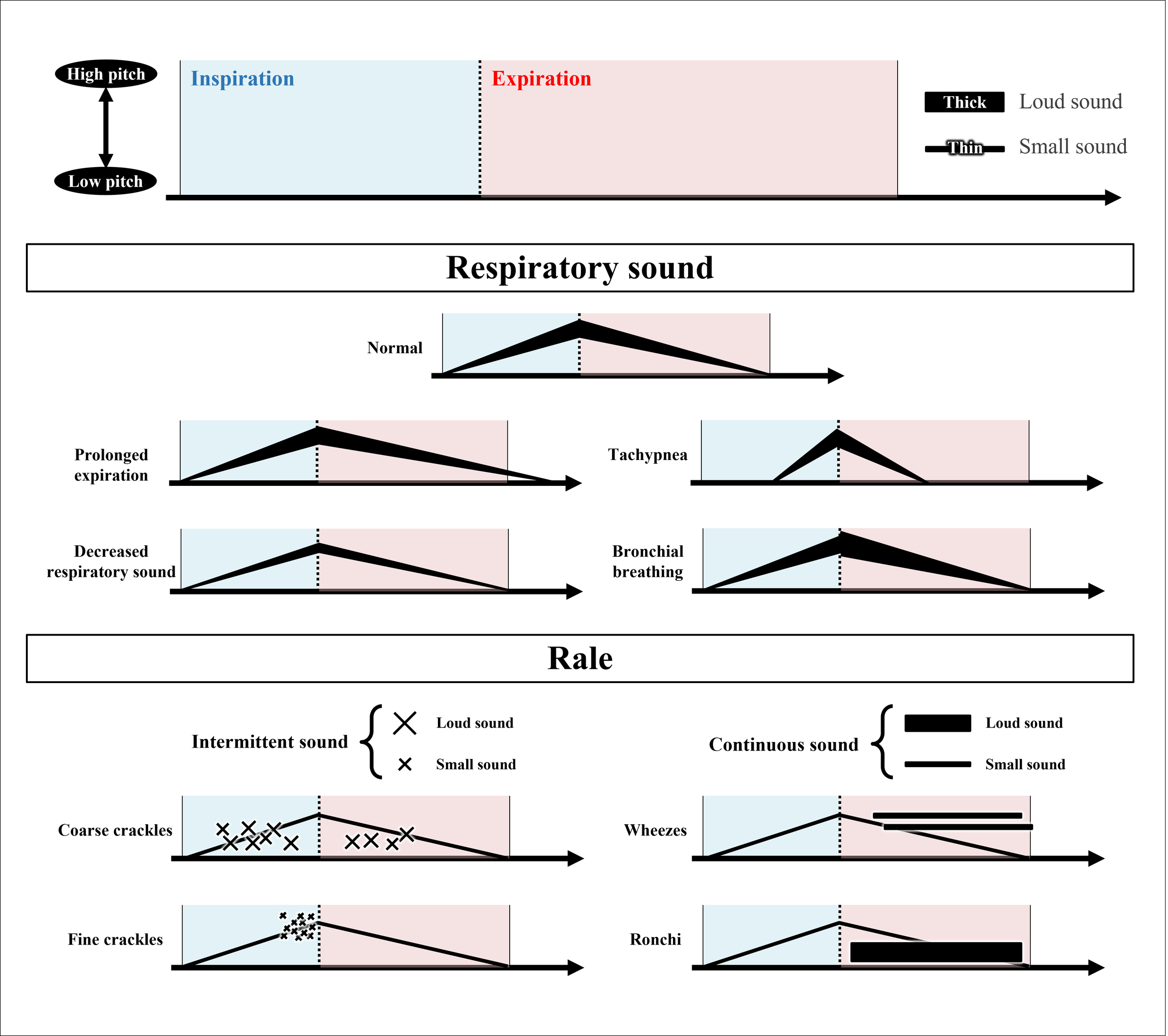

Supplement: S5 Fig — (TIF) [file pone.0282337.s005.tif]
