# Supplementary material for: The effects of simple graphical and mental visualization of lung sounds in teaching lung auscultation during clinical clerkship: A preliminary study
Source: PLoS One. 2023 Mar 17;18(3):e0282337. doi: 10.1371/journal.pone.0282337 (PMC10022769; doi:10.1371/journal.pone.0282337)
Supplement: S1 Table — (DOCX) [file pone.0282337.s006.docx]

**Supplementary table 1. Questionnaire items before and after the training on lung auscultation.**

| **Before the CC in Respiratory Medicine** | **Responses** |
| --- | --- |
| (A1) How is your current lung sound auscultation ability? | Five-point Likert scale  1 (Not confident [cannot listen at all]) to 5 (Confident [able to listen and distinguish between lung sounds and rales]) |
| (A2) Have you ever heard each lung sound from a patient?  Decreased respiratory sounds, bronchial breathing, prolonged expiration, coarse crackles, fine crackles, wheezes, rhonchi, squawk, and pleural friction rub | Yes or No |
| (A3) How confident are you in auscultating each lung sound?  Same items as in question A2. | The response options are same as in question A1. |
| **After the CC in Respiratory Medicine** |  |
| (B1) How is your current lung sound auscultation ability? | The response options are same as in question A1. |
| (B2) Have you ever heard each lung sound from a patient?  Same items as in question A2. | The response options are same as in question A2. |
| (B3) How confident are you in auscultating each lung sound?  Same items as in question A2. | The response options are same as in question A1. |
| (B4) What is your level of satisfaction with the education program on lung auscultation? | Five-point Likert scale  1 (extremely poor) to 5 (extremely good) |

CC, clinical clerkship.
