# Supplementary material for: The effects of simple graphical and mental visualization of lung sounds in teaching lung auscultation during clinical clerkship: A preliminary study
Source: PLoS One. 2023 Mar 17;18(3):e0282337. doi: 10.1371/journal.pone.0282337 (PMC10022769; doi:10.1371/journal.pone.0282337)
Supplement: S2 Table — (DOCX) [file pone.0282337.s007.docx]

**Supplementary table 2.** *P*-value in the test of normality of each parameter (*n* = 65).

|  | Visualization group (*n* = 35) | Control group  (*n* = 30) |
| --- | --- | --- |
| Satisfaction | ＜0.001 | ＜0.001 |
| Confidence |  |  |
| Pre-questionnaire | ＜0.001 | 0.010 |
| Post-questionnaire | ＜0.001 | ＜0.001 |
| Score of lung auscultation |  |  |
| Pre-test | 0.008 | 0.610 |
| Post-test | 0.049 | 0.747 |
