# Supplementary material for: The effects of simple graphical and mental visualization of lung sounds in teaching lung auscultation during clinical clerkship: A preliminary study
Source: PLoS One. 2023 Mar 17;18(3):e0282337. doi: 10.1371/journal.pone.0282337 (PMC10022769; doi:10.1371/journal.pone.0282337)
Supplement: Attachment — Submitted filename: PONE-D-22-25852.pdf [file pone.0282337.s009.pdf]

PLOS ONE

Does simple graphical and mental visualization of lung sounds improve the auscultation skills of clinical clerkship students?

--Manuscript Draft--

**Manuscript Number:**	PONE-D-22-25852
**Article Type:**	Research Article
**Full Title:**	Does simple graphical and mental visualization of lung sounds improve the auscultation skills of clinical clerkship students?
**Short Title:**	Visualization of lung sounds in clinical clerkship
**Corresponding Author:**	Hajime Kasai, M.D.<br>Graduate School of Medicine, Chiba University<br>Chiba, JAPAN
**Keywords:**	Clinical skills; Undergraduate; General; Methods
**Abstract:**	Introduction<br><br>The study was conducted to evaluate the effects of visualization-based training on lung auscultation during clinical clerkship (CC) in the department of Respiratory Medicine on student skills and confidence.<br><br>Methods<br><br>The study period was December 2020 to November 2021. In total, 79 students attended a lecture on lung auscultation featuring a simulator (Mr. Lung™). Out of these attendees, 35 students (visualization group) received additional training wherein they were asked to mentally visualize lung sounds using a diagram of graphical visualized lung sounds as an example. All students answered questions on their self-efficacy regarding lung auscultation before and after four weeks of CC. They also took a lung auscultation test with the simulator at the beginning of CC (pre-test) and on the last day of the third week (post-test) (maximum score: 25). We compared the answers in the questionnaire and the test scores between the visualization group and students who only attended the lecture (control group, n=30).<br><br>Results<br><br>Confidence in auscultation of lung sounds significantly increased in both groups (five-point Likert scale, visualization group: pre 1.5±0.1 to post 3.2±0.1, p<0.001; control group: 1.7±0.1 to 2.9±0.1, p<0.001), and was significantly higher in the visualization group than in the control group. Moreover, test scores increased in both groups (visualization group: pre-test 11.4±0.5 to post-test 15.1±0.6, p<0.001; control group: 11.1±0.5 to 14.3±0.6, p<0.001). Although there were no differences between the pre- and post-tests scores of both groups, the score of the visualization group tended to increase compared with that of the control group (p=0.623).<br><br>Conclusion<br><br>Visualizing lung sounds may increase medical students' confidence in their lung auscultation skills; notably, this may reduce their resistance to lung auscultation and encourage the repeated auscultation necessary to further improve their long-term auscultation abilities over the long term.
**Order of Authors:**	Hajime Kasai, Ph.D
	Ayaka Kuriyama
	Kiyoshi Shikino
	Yuki Shiko
	Chiaki Kawame

Powered by Editorial Manager® and ProduXion Manager® from Aries Systems Corporation

	Kenichiro Takeda
	Nami Hayama
	Takuji Suzuki
	Shoichi Ito, Ph.D
**Additional Information:**	
**Question**	**Response**
**Financial Disclosure**<br><br>Enter a financial disclosure statement that describes the sources of funding for the work included in this submission. Review the submission guidelines for detailed requirements. View published research articles from *PLOS ONE* for specific examples.<br><br>This statement is required for submission and **will appear in the published article** if the submission is accepted. Please make sure it is accurate.<br><br>**Unfunded studies**<br>Enter: *The author(s) received no specific funding for this work.*<br><br>**Funded studies**<br>Enter a statement with the following details:<br>• Initials of the authors who received each award<br>• Grant numbers awarded to each author<br>• The full name of each funder<br>• URL of each funder website<br>• Did the sponsors or funders play any role in the study design, data collection and analysis, decision to publish, or preparation of the manuscript?<br>• **NO** - Include this sentence at the end of your statement: *The funders had no role in study design, data collection and analysis, decision to publish, or preparation of the manuscript.*<br>• **YES** - Specify the role(s) played.<br><br>* typeset	The author(s) received no specific funding for this work.
**Competing Interests**<br><br>Use the instructions below to enter a competing interest statement for this submission. On behalf of all authors, disclose any competing interests that	The authors have declared that no competing interests exist.

could be perceived to bias this work—acknowledging all financial support and any other relevant financial or non-financial competing interests.<br><br>This statement is required for submission and **will appear in the published article** if the submission is accepted. Please make sure it is accurate and that any funding sources listed in your Funding Information later in the submission form are also declared in your Financial Disclosure statement.<br><br>View published research articles from *PLOS ONE* for specific examples.<br><br>**NO authors have competing interests**<br><br>Enter: *The authors have declared that no competing interests exist.*<br><br>**Authors with competing interests**<br><br>Enter competing interest details beginning with this statement:<br><br>*I have read the journal's policy and the authors of this manuscript have the following competing interests: [insert competing interests here]*<br><br>* typeset	
**Ethics Statement**<br><br>Enter an ethics statement for this submission. This statement is required if the study involved:<br><br>• Human participants<br>• Human specimens or tissue<br>• Vertebrate animals or cephalopods<br>• Vertebrate embryos or tissues<br>• Field research<br><br>Write "N/A" if the submission does not require an ethics statement.	This study was approved by the ethics committee of Chiba University (approval number 3425).

Powered by Editorial Manager® and ProduXion Manager® from Aries Systems Corporation

General guidance is provided below. Consult the submission guidelines for detailed instructions. **Make sure that all information entered here is included in the Methods section of the manuscript.**<br><br>**Format for specific study types**<br><br>**Human Subject Research (involving human participants and/or tissue)**<br>• Give the name of the institutional review board or ethics committee that approved the study<br>• Include the approval number and/or a statement indicating approval of this research<br>• Indicate the form of consent obtained (written/oral) or the reason that consent was not obtained (e.g. the data were analyzed anonymously)<br><br>**Animal Research (involving vertebrate animals, embryos or tissues)**<br>• Provide the name of the Institutional Animal Care and Use Committee (IACUC) or other relevant ethics board that reviewed the study protocol, and indicate whether they approved this research or granted a formal waiver of ethical approval<br>• Include an approval number if one was obtained<br>• If the study involved *non-human primates*, add *additional details* about animal welfare and steps taken to ameliorate suffering<br>• If anesthesia, euthanasia, or any kind of animal sacrifice is part of the study, include briefly which substances and/or methods were applied<br><br>**Field Research**<br><br>Include the following details if this study involves the collection of plant, animal, or other materials from a natural setting:<br>• Field permit number<br>• Name of the institution or relevant body that granted permission	
**Data Availability**<br><br>Authors are required to make all data	Yes - all data are fully available without restriction

Powered by Editorial Manager® and ProduXion Manager® from Aries Systems Corporation

underlying the findings described fully available, without restriction, and from the time of publication. PLOS allows rare exceptions to address legal and ethical concerns. See the PLOS Data Policy and FAQ for detailed information.<br><br>A Data Availability Statement describing where the data can be found is required at submission. Your answers to this question constitute the Data Availability Statement and **will be published in the article**, if accepted.<br><br>**Important:** Stating 'data available on request from the author' is not sufficient. If your data are only available upon request, select 'No' for the first question and explain your exceptional situation in the text box.<br><br>Do the authors confirm that all data underlying the findings described in their manuscript are fully available without restriction?	
**Describe where the data may be found in full sentences. If you are copying our sample text, replace any instances of XXX with the appropriate details.**<br><br>• If the data are **held or will be held in a public repository**, include URLs, accession numbers or DOIs. If this information will only be available after acceptance, indicate this by ticking the box below. For example: *All XXX files are available from the XXX database (accession number(s) XXX, XXX.*).<br>• If the data are all contained **within the manuscript and/or Supporting Information files**, enter the following: *All relevant data are within the manuscript and its Supporting Information files.*<br>• If neither of these applies but you are able to provide **details of access elsewhere**, with or without limitations, please do so. For example:<br><br>*Data cannot be shared publicly because of [XXX]. Data are available from the*	All relevant data are within the manuscript and its Supporting Information files.

XXX Institutional Data Access / Ethics Committee (contact via XXX) for researchers who meet the criteria for access to confidential data.<br><br>*The data underlying the results presented in the study are available from (include the name of the third party and contact information or URL).*<br>• This text is appropriate if the data are owned by a third party and authors do not have permission to share the data.<br><br>* typeset	
Additional data availability information:	Tick here if the URLs/accession numbers/DOIs will be available only after acceptance of the manuscript for publication so that we can ensure their inclusion before publication.

Powered by Editorial Manager® and ProduXion Manager® from Aries Systems Corporation

Click here to access/download;Manuscript;Manuscript_0915.docx

Does simple graphical and mental visualization of lung sounds improve the auscultation skills of clinical clerkship students?

Running Head: Visualization of lung sounds in Cc

Authors: Ayaka Kuriyama[1*], Hajime Kasai[1,2,3*], Kiyoshi Shikino[4], Yuki Shiko[5], Chiaki Kawame[1], Kenichiro Takeda[1], Nami Hayama[1], Takuji Suzuki[1], Shoichi Ito[2,3]

[1] *Department of Respirology, Chiba University Graduate School of Medicine, Chiba, Japan*

[2] *Health Professional Development Center, Chiba University Hospital, Chiba, Japan*

[3] *Department of Medical Education, Chiba University Graduate School of Medicine, Chiba, Japan*

[4] *Department of General Medicine, Graduate School of Medicine, Chiba University, Chiba, Japan*

[5] *Biostatistics Section, Clinical Research Center, Chiba University Hospital*

[*]*Contributed equally*

Corresponding author: Hajime Kasai

Department of Respirology, Chiba University Graduate School of Medicine

1-8-1 Inohana, Chuo-ku, Chiba-shi, Chiba 260-8670, Japan

TEL: +81-43-222-7171 Ext. 71014

FAX: +81-43-226-2176

E-mail: daikasai6075@yahoo.co.jp

Abstract

Introduction: The study was conducted to evaluate the effects of visualization-based training on lung auscultation during clinical clerkship (CC) in the department of Respiratory Medicine on student skills and confidence.

Methods: The study period was December 2020 to November 2021. In total, 79 students attended a lecture on lung auscultation featuring a simulator (Mr. Lung™). Out of these attendees, 35 students (visualization group) received additional training wherein they were asked to mentally visualize lung sounds using a diagram of graphical visualized lung sounds as an example. All students answered questions on their self-efficacy regarding lung auscultation before and after four weeks of CC. They also took a lung auscultation test with the simulator at the beginning of CC (pre-test) and on the last day of the third week (post-test) (maximum score: 25). We compared the answers in the questionnaire and the test scores between the visualization group and students who only attended the lecture (control group, n=30).

Results: Confidence in auscultation of lung sounds significantly increased in both groups (five-point Likert scale, visualization group: pre 1.5±0.1 to post 3.2±0.1, p<0.001; control group: 1.7±0.1 to 2.9±0.1, p<0.001), and was significantly higher in the visualization group than in the control group. Moreover, test scores increased in both groups (visualization group: pre-test 11.4±0.5 to post-test 15.1±0.6, p<0.001; control group: 11.1±0.5 to 14.3±0.6, p<0.001). Although there were no differences between the pre- and post-tests scores of both groups, the score of the visualization group tended to increase compared with that of the control group (p=0.623).

Conclusion: Visualizing lung sounds may increase medical students' confidence in their lung auscultation skills; notably, this may reduce their resistance to lung auscultation and encourage the repeated auscultation necessary to further improve their long-term auscultation abilities over the long term.

Keywords:

Clinical skills, Undergraduate, General, Methods

Keywords (user): auscultation; lung sounds; visualization

Introduction

Lung auscultation is a fundamental part of physical examination; it is routinely performed by doctors and other medical professionals alike. Lung auscultation is a useful non-invasive method for clinical reasoning and identifying a respiratory condition using only a stethoscope [1]. Regarding clinical respiratory examination skills, auscultation of lung sounds is often learned during medical school [2]. However, it is not easy to accurately listen to lung sounds and assess respiratory condition. In a study comparing the auscultation skills of medical students and physicians, there was no difference in skill, except for pulmonologists [3, 4]. This finding suggests that physicians may not have better lung auscultation skills than medical students.

Recent advances in digital stethoscopes have made it possible to record lung sounds and share them among multiple medical professionals, as well as to analyze lung sounds automatically, perform auscultation wirelessly, and provide telemedicine [5]. However, effective education for lung auscultation has not been developed. The skills of lung auscultation have been taught using simulators in addition to textbooks and audio sources [6]. Moreover, in recent years, it has become possible to listen to lung sounds through internet video-sharing sites and smartphone applications [7–9]. Medical students are often trained in auscultation at school, and after becoming doctors, may find on-the-job training to be their main learning opportunity for lung auscultation. Bernardi et al. examined the effects of simulator-based education on lung sounds and heart sounds and observed an improvement in the ability of auscultation of heart sounds, but no change in that of lung sounds [10]. The reason for this result could be that the participants were asked to choose the relevant lung sounds from a list of options, while the heart sounds were answered graphically. Therefore, the addition of visual information regarding lung sounds may improve lung auscultation learning.

Lung sounds have been visualized by spectrograms, which have been published in textbooks, with the vertical axis representing sound pitch and the horizontal axis representing time [11]. In the spectrogram, intermittent rales are represented by short vertical lines and continuous rales are represented by horizontal lines. While it is useful to learn the characteristics of lung sounds [12], spectrograms are not available in all institutions, and it is difficult to visualize them with a personal stethoscope.

Herein, we created a simple graphical and mental visualization of lung sounds using a combination of lines and circles in lung auscultation. This study, therefore, aimed to evaluate the effect of educational methods of lung sound auscultation using visualization for medical students in clinical clerkship (CC).

Materials and Methods

Setting

Pre-clerkship course and CC

In Chiba University's school of medicine, each grade comprises around 120 students. Medical schools in Japan offer a six-year curriculum, with the final two years generally spent in CCs [13]. In our university, the CC starts in December of the fourth year and ends in October of the sixth year. Students are rotated from one department to the other every four weeks. In the third year, before the CC, students attended a one-hour lecture on lung auscultation using a simulator. Subsequently, each student performed self-learning using a textbook and/or the simulator in preparation for the objective structured clinical examination (OSCE), which is administered in the fall of the fourth year before CC.

CC in the Respiratory Medicine department and study samples

In our department's CC, groups of seven to eight medical students underwent a four-week training program as members of a medical team of doctors and residents. All medical students were assigned two to four patients during this four-week period and performed daily physical examinations, including auscultation of the lungs.

A total of 89 medical students underwent CC in Respiratory Medicine at Chiba University Hospital between December 2020 and November 2021. Informed consent was procured during CC orientation from the participants for use of participants' test results regarding lung auscultation as well as their questionnaire responses. Students who did not answer all parts of the questionnaire or take the lung auscultation test were excluded from the study. The 18 students who practiced in February and September 2021, during the COVID-19 pandemic in Japan, were also excluded, since the lectures and tests could not be held due to hospital access restrictions.

This study was approved by the ethics committee of Chiba University (approval number 3425). The study database was anonymized.

Procedure of education on lung sound auscultation with visualization

The lecture on lung auscultation lasted for an hour and was presented to all students on the first day of CC in Respiratory Medicine. It consisted of an explanation of the mechanism of hearing lung sounds and diseases, and auscultation of various lung sounds by students with the simulator (Mr. Lung®, Kyoto-Kagaku, Kyoto). Mr. Lung® is a mannequin-type lung sounds auscultation simulator widely used in medical schools in Japan [14].

The medical students were divided into two groups: a visualization group in which the participants attended the lecture and visualized the lung sounds, and a control group in which students only attended the lecture (Figure 1). The two groups were formulated on a rotational

basis (Visualization group: January, April, June, October, November; Control group: December, March, May, July).

Fig 1 Flow chart of the participants' selections and groups.

The students in the visualization group received additional training using a diagram of graphically visualized lung sounds, as shown in Figure 2. In the diagram, the vertical axis represents the pitch of lung sounds, and the horizontal axis represents the duration of lung sounds. For respiratory sounds, the thickness of the line represents the loudness of the sound. For the intermittent rales, each crackle is represented by a circle, and the height of the circle's position indicates the pitch of the sound, while the size of the circle indicates the loudness of the sound. Coarse crackles are presented as circles which are drawn at a lower position on inspiratory and/or expiratory. Fine crackles are presented as circles that are drawn at the end of the inspiratory and are concentrated at a high position. For continuous rales, a horizontal bar was used. The height of the horizontal bar position indicates the pitch of the sound, and the thickness of the bar indicates the loudness of the sound. Wheezes are mainly represented by a horizontal bar at the high end of the expiration, and rhonchi are also mainly represented by a horizontal bar at the low end of the expiration.

Fig 2 The graphical visualization of lung sounds using a combination of lines and circles. (A diagram showing inspiration and expiration was used to visualize the lung sounds. In the diagram, the vertical axis represents the pitch of lung sounds and the horizontal axis represents the duration of lung sounds. For respiratory sounds, the thickness of the line represents the loudness of the sound. For the intermittent rales, each crackle is represented by a circle, the height of position of the circle indicates the pitch of the sound, and the size of the circle

indicates the loudness of the sound. Coarse crackles are presented as circles that are drawn at a lower position on inspiratory and/or exspiratory. Fine crackles are presented as circles that are drawn at the end of the inspiratory and are concentrated at a high position. For continuous rales, a horizontal bar was used. The height of position of the horizontal bar indicates the pitch of the sound and the thickness of the bar indicates the loudness of the sound. Wheezes are mainly represented by a horizontal bar at the high end of the expiration and rhonchi are also mainly represented by a horizontal bar at the low end of the expiration.)

During the lecture, the students drew the various lung sounds they heard on a blank diagram. Then, the students were instructed to mentally visualize lung sounds while performing lung auscultation for patients.

Evaluation of the effects of education on lung sound auscultation with visualization

Questionnaire

At the beginning and end of the department’s CC, students responded to the following questions on their experience and confidence with lung auscultation: (1) How is your current lung sound auscultation ability? (2) Have you ever heard each lung sound from a patient? (3) How confident are you in each of the lung sounds? Questions 1 and 3 were rated on a five-point Likert scale, with scores ranging from 1 (Not confident [cannot listen at all]) to 5 (Confident [able to listen and distinguish between lung sounds and rales]). Moreover, for questions 2 and 3, the students responded to each of the following: decreased respiratory sounds, bronchial breathing, prolonged expiration, coarse crackles, fine crackles, wheezes, rhonchi, squawk, and pleural friction rub. The classification of lung sounds was based on a

review by Bohadana et al. [15]. In addition to the above three questions, the students reported their satisfaction levels with the education on lung auscultation at the end of CC in Respiratory Medicine.

Fig 3 Protocol for lung auscultation education and questionnaire and test regarding lung auscultation.

Performance of lung auscultation

Students' lung auscultation performance was evaluated by an auscultation test of 10 cases using the Mr. Lung™ simulator. The pre-test was performed at the beginning of CC in Respiratory Medicine before the lecture. The post-test was performed on the last day of the third week of the CC. The pre- and post-tests consisted of the same 10 sounds, while each case was given in a different order (Table 1). In each case, one point was given for each correct answer of lung sound abnormality and location. In four cases, in which more than one abnormal lung sound could be heard, one point was given for each sound. Therefore, the maximum score was 25 points. Both the pre-test and post-test taken by the control group were multiple-choice forms (Supplemental Figure 1A). The pre-test taken by the visualization group was a multiple-choice form, while the post-test was a multiple-choice form that included a space to draw a figure where there was an abnormality (Supplemental Figure 1B).

Table 1 Abnormal lung sounds and where they were listened, as used in the lung auscultation examination

Case of abnormal lung sounds	Location
Decreased breath sound	Left lower lung field

Coarse crackles	Right upper middle lung field
Coarse crackles	Bilateral upper lung fields
Fine crackles	Bilateral lower pulmonary region
Fine crackles	Bilateral whole lung fields
Wheezes, Prolonged expiration	Bilateral upper lung fields
Ronchi, Prolonged expiration	Bilateral upper lung fields
Wheezes, Ronchi, Prolonged expiration	Bilateral upper lung fields
Squawk, Coarse crackles	Right upper and middle lung fields
Pleural friction rub	Right middle and lower lung fields

The pre- and post-tests consisted of the same ten sounds, while each case was given in a different order.

We then compared the answers in the questionnaire and the test scores between the visualization group and control group.

Statistical analysis

All results are expressed in terms of mean ± standard deviation (SD), unless otherwise indicated. The Wilcoxon signed-rank test was used to compare the answers before and after our education. Confidence in and scores on lung auscultation in the two groups before and after our lecture were compared using analysis of covariance (ANCOVA), by adjusting the values of the pre-questionnaire and pre-test results for each group. A p-value < 0.05 was considered statistically significant. All statistical analyses were performed using JMP 15.0 software (Cary, North Carolina, USA) and SAS software version 9.4 (SAS Institute, Cary, USA).

Results

In total, 71 students completed the lecture for auscultation of lung sounds. Of these students, three did not take the post-test and were therefore excluded from the study. Another three students were also excluded because their questionnaires had insufficient data. Finally, 65 students were included in the study (Figure 1).

Questionnaire

Based on an analysis of their responses, students' satisfaction level with our education was high in both groups, but slightly higher in the visualization group (visualization group: 4.5±0.1 vs. control group: 4.2±0.1, $p = 0.150$). There was no significant difference between the two groups in the experience of listening to each lung sound at the beginning and end of CC in Respiratory Medicine (Supplemental Figure 2).

Confidence in lung auscultation significantly increased in both groups (five-point Likert scale; visualization group, pre-test: 1.5±0.6 to post-test: 3.3±0.7, $p < 0.001$; control group, 1.7±0.6 to 2.9±0.8, $p < 0.001$). Confidence in lung auscultation after CC was found to be significantly higher in the visualization group than in the control group ($p = 0.028$; adjusted mean difference between pre- and post-test, 1.7±0.1 vs. 1.3±0.1, $p = 0.020$). In contrast, there was no difference between the two groups in self-assessment of listening to each lung sound (Supplemental Figure 3).

Results of tests of lung auscultation

The test score increased in both groups (visualization group, pre- 11.4±2.5 to post-test 15.1±3.5, $p < 0.001$; control group, 11.1±3.3 to 14.3±3.2, $p < 0.001$) as shown in Table 2. Although there was no difference between the scores of the pre- and post-tests of both groups

(pre-test, $p = 0.827$; post-test, $p = 0.290$), the visualization group scores tended to increase as compared to that of the control group (adjusted mean difference between pre- and post-tests, 3.7±0.5 vs. 3.1±0.6, $p = 0.424$).

Table 2 Changes in the confidence level pertaining to lung auscultation and the score of lung auscultation before and after education (*n* = 65).

	Visualization group (n = 35)	Control group (n = 30)	p-value
Confidence in lung auscultation, mean (SD)			
Pre-questionnaire	1.5 (0.6)	1.7 (0.6)	0.128
Post-questionnaire	3.3 (0.7)	2.9 (0.8)	**0.028**
Mean difference	1.8 (0.7)	1.2 (1.0)	**0.005**
Adjusted mean difference, least mean square (SE)	1.7 (0.1)	1.3 (0.1)	**0.020***
Score of lung auscultation, mean (SD)			
Pre-test	11.4 (2.5)	11.1 (3.3)	0.827
Post-test	15.1 (3.5)	14.3 (3.2)	0.290
Mean difference	3.7 (3.4)	3.2 (3.6)	0.623
Adjusted mean difference, least mean square (SE)	3.7 (0.5)	3.1 (0.6)	0.424*

* Analysis of covariance (ANCOVA) was performed and pre-score was adjusted.

(SD: standard deviation; SE: standard error)

Discussion

To the best of our knowledge, this is the first study to evaluate the effect of graphical and mental visualization of lung sounds in lung auscultation teaching during CC. There are two main findings. First, the visualization of lung sounds can improve medical students' confidence in their lung auscultation. Second, the visualization of lung sounds may improve medical students' ability to auscultate lung sounds, although the effect is limited. Our method does not require any special tools and can be easily and quickly implemented into usual education.

The visualization of lung sounds in lung auscultation can boost medical students' confidence in lung auscultation and help develop their lung auscultation skills. In the auscultation of heart sounds, the visualization of the timing of rhythm and murmur has been used [16]. Lung sounds have often been visualized in textbooks and reports [1]. Sestini et al. reported that a multimedia presentation of acoustic and graphic characteristics of lung sounds could improve lung auscultation learning among medical students. In their study, the questionnaire completed by the students indicated that the association of the acoustic signals with their visual image was useful for learning and understanding lung sounds [12]. However, there have been few opportunities to visualize lung sounds in daily practice. In addition to lectures using spectrograms and sound sources as presented in the report, we evaluated how simple graphical and mental visualization of lung sounds affected the lung auscultation of medical students in CC. Although the primary objective was to identify the lung sound abnormalities that were present in the patient, limited interest has been shown in determining the timing of breathing and/or the pitch of the abnormal sounds that can be heard. However, it is important to recognize the timing of breathing and the pitch of abnormal lung sounds for the evaluation of the respiratory status and for clinical reasoning. Moreover, there are four learning styles: visual, auditory, reading/writing, and kinesthetic [17]. The acquisition of auscultation

skills can basically be referred to as an auditory style of learning. Visualization of lung sounds can make lung auscultation learning multimodal by combining visual and auditory styles and this may help the students to be more aware of the timing and pitch of lung sounds. The visualization group showed greater confidence in lung auscultation, supporting this possibility. Since post-test scores were only around half the maximum number of points, only having confidence in lung auscultation will not suffice. However, the satisfaction level was also significantly higher in the visualization group, suggesting that visualization may have facilitated students' better understanding of lung sounds. Moreover, without confidence in lung auscultation, students may be hesitant to perform lung auscultation on patients. There is a possibility that students who are more confident in lung auscultation actively repeat lung auscultation more often. The repetitive practice of clinical skills can contribute to the retention of these skills [18]. In these students, auscultation skills may improve further in the long term.

The effect on the improvement score of lung auscultation was limited. Although there was no significant difference between the post-test scores of both groups, the visualizing group's scores tended to improve more than that of the control group. There are several possible reasons for the limited effect of our educational method. During the lecture, we presented a visualization of lung sounds and asked the students to draw a lung sound which they heard during the lecture. Then, the students were also instructed to imagine lung sounds during their daily practice of lung auscultation. However, it was not clear how often the students continued to visualize lung sounds in their daily practice. Instruction to repeatedly visualize lung sounds may be insufficient. It is possible that retention by instructing the students to repeatedly visualize lung sounds during their daily practice may have been more effective. Additional opportunities to draw lung sounds using the simulator or recorded lung sounds may have enhanced the effectiveness of our educational method; this possibility, along with the aforementioned one, could be addressed by future research. Furthermore, it is not clear how

the medical students actually heard the lung sounds from their answers that a lung sound abnormality was heard. By providing regular opportunities to capture lung sounds that can be heard using our method, it will be possible to clarify the gap between the findings of medical students and those of the supervising physician. As a result, lung auscultation can be effectively taught. In any case, our educational method of lung sound visualization does not require any additional tools, nor does it require much effort to implement. Although its effectiveness may be limited, it is a method that can be easily introduced into regular education and practice.

Study limitations

The present study has three main limitations. First, as it was performed at just one university in Japan, the number of students was small. Second, the experience of lung auscultation in other departments, the diseases of the cases that each student oversaw during CC in Respiratory Medicine, and each student's learning status may have influenced the results. Third, long-term effects have not been explored in our study.

Conclusion

The visualization of lung sounds may improve medical students' lung auscultation skills and their confidence in auscultation. Although the effect of lung sound visualization is limited, it can be undertaken without additional devices such as special simulators and serve as a useful educational strategy. Further improvement in teaching methods may increase the learning effect of visualization.

References

1. Sarkar M, Madabhavi I, Niranjan N, Dogra M. Auscultation of the respiratory system. Ann Thorac Med. 2015;10: 158-168. doi: 10.4103/1817-1737.160831.
2. Kerzner S, Mao RQ, Patel JN, Sreeraman S, Profetto J. Identifying the gap in clinical skills: a pilot study investigating the use of clinical respiratory examination skills in practice. Educ Prim Care. 2021;32: 259-265. doi: 10.1080/14739879.2021.1901614.
3. Mangione S, Nieman LZ. Pulmonary auscultatory skills during training in internal medicine and family practice. Am J Respir Crit Care Med. 1999;159: 1119-1124. doi: 10.1164/ajrccm.159.4.9806083.
4. Hafke-Dys H, Bręborowicz A, Kleka P, Kociński J, Biniakowski A. The accuracy of lung auscultation in the practice of physicians and medical students. PLOS ONE. 2019;14: e0220606. doi: 10.1371/journal.pone.0220606.
5. Kim Y, Hyon Y, Lee S, Woo SD, Ha T, Chung C. The coming era of a new auscultation system for analyzing respiratory sounds. BMC Pulm Med. 2022;22: 119. doi: 10.1186/s12890-022-01896-1.
6. Kaminsky J, Bianchi R, Eisner S, Ovitsh R, Lopez AM, Smith L, et al. Respiratory auscultation lab using a cardiopulmonary auscultation simulation manikin. MedEdportal. 2021;17: 11107. doi: 10.15766/mep_2374-8265.11107.
7. Practical clinical skills. Lung sounds | Breath sounds. [Cited Sep. 5 2022]. Available from: https://www.practicalclinicalskills.com/lung-sounds.
8. Thinklabs-YouTube. [Cited Sep. 5 2022]. Available from: https://www.youtube.com/c/thinklabs1.
9. Fraiwan M, Fraiwan L, Khassawneh B, Ibnian A. A dataset of lung sounds recorded from the chest wall using an electronic stethoscope. Data Brief. 2021;35: 106913. doi: 10.1016/j.dib.2021.106913.

10. Bernardi S, Giudici F, Leone MF, Zuolo G, Furlotti S, Carretta R, et al. A prospective study on the efficacy of patient simulation in heart and lung auscultation. BMC Med Educ. 2019;19: 275. doi: 10.1186/s12909-019-1708-6.

11. Bae W, Kim K, Yoon JS. Interrater reliability of spectrogram for detecting wheezing in children. Pediatr Int. 2021;64: e15003. doi: 10.1111/ped.15003.

12. Sestini P, Renzoni E, Rossi M, Beltrami V, Vagliasindi M. Multimedia presentation of lung sounds as a learning aid for medical students. Eur Respir J. 1995;8: 783–788.

13. Kozu T. Medical education in Japan. Acad Med. 2006;81: 1069-1075. doi: 10.1097/01.ACM.0000246682.45610.dd.

14. Arimura Y, Komatsu H, Yanagi S, Matsumoto N, Okayama A, Hayashi K, et al. [Educational usefulness of lung auscultation training with an auscultation simulator]. Nihon Kokyuki Gakkai Zasshi. 2011;49: 413-418.

15. Bohadana A, Izbicki G, Kraman SS. Fundamentals of lung auscultation. N Engl J Med. 2014;370: 744-751. doi: 10.1056/NEJMra1302901.

16. Reed NE, Nie Y, Mahnke CB. A portable graphical representation tool for phonocardiograms. Annu Int Conf IEEE Eng Med Biol Soc. Annu Int Conf IEEE Eng Med Biol Soc. 2009;2009: 3111-3114. doi: 10.1109/IEMBS.2009.5332544.

17. Fleming ND, Mills C. Not another inventory, rather a catalyst for reflection. To Improve Acad. 1992;11: 137-155. doi: 10.1002/j.2334-4822.1992.tb00213.x.

18. Offiah G, Ekpotu LP, Murphy S, Kane D, Gordon A, O’Sullivan M, et al. Evaluation of medical student retention of clinical skills following simulation training. BMC Med Educ. 2019;19: 263. doi: 10.1186/s12909-019-1663-2.

Supporting information

Supplementary Figure 1. Answer sheet for lung sound auscultation test.

A multiple-choice form (A) was used as the pre-test for the control and visualization group and the post-test for the control group. A multiple-choice form that combined a space in which to draw a figure where there was an abnormality (B) was used as the post-test for the visualization group.

Supplementary Figure 2. The responses of the visualization group (*n* = 35) and the control group (*n* = 30) regarding the experience of listening to each lung sound. C, control group; NS, not significant; V, visualization group

Supplementary Figure 3. The responses of the visualization group (*n* = 35) and the control group (*n* = 30) regarding self-assessment of listening to each lung sound.

 Click here to access/download;Figure;Figure 1.png

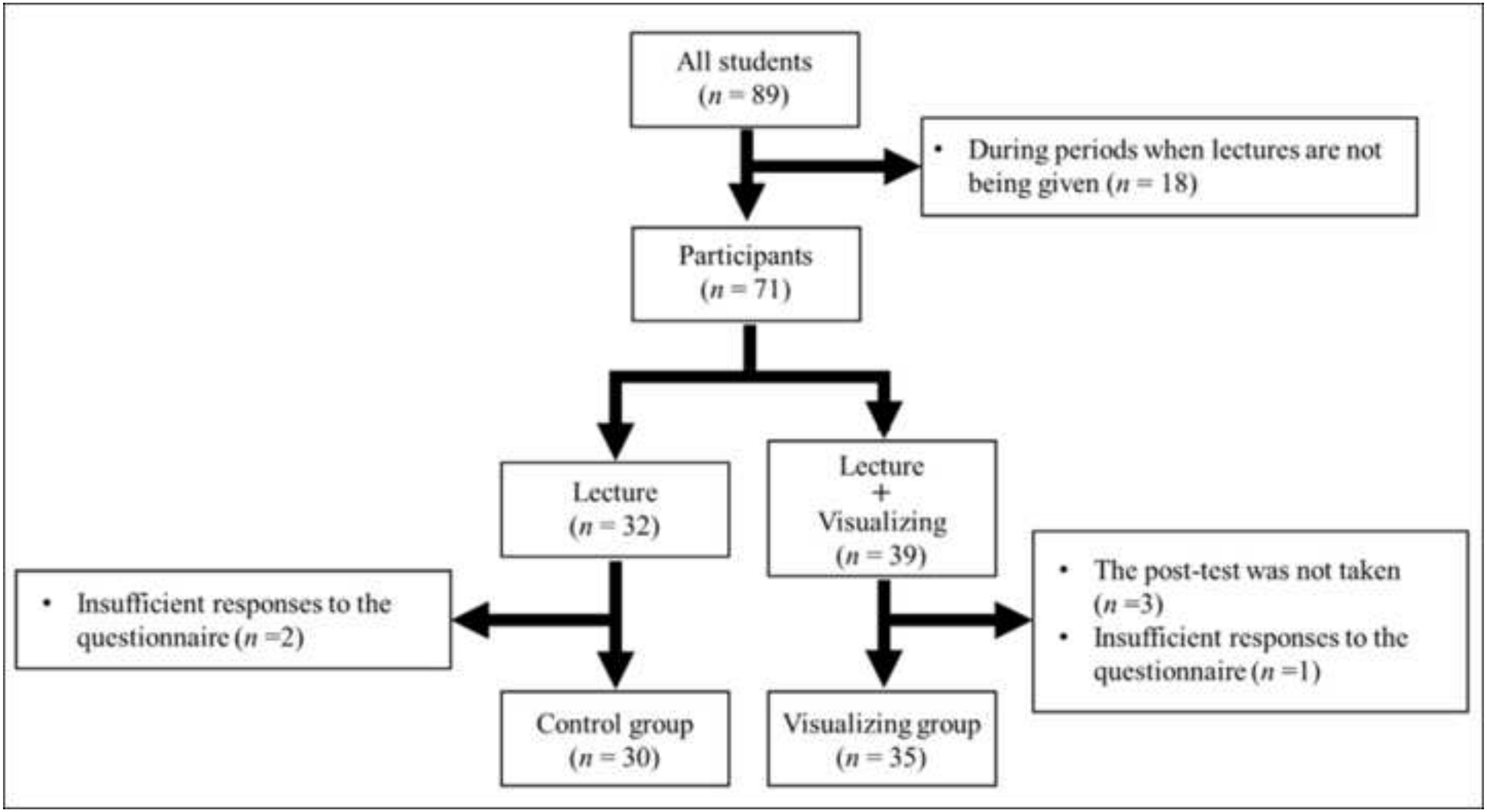

Figure 2

Click here to access/download;Figure;Figure 2.png

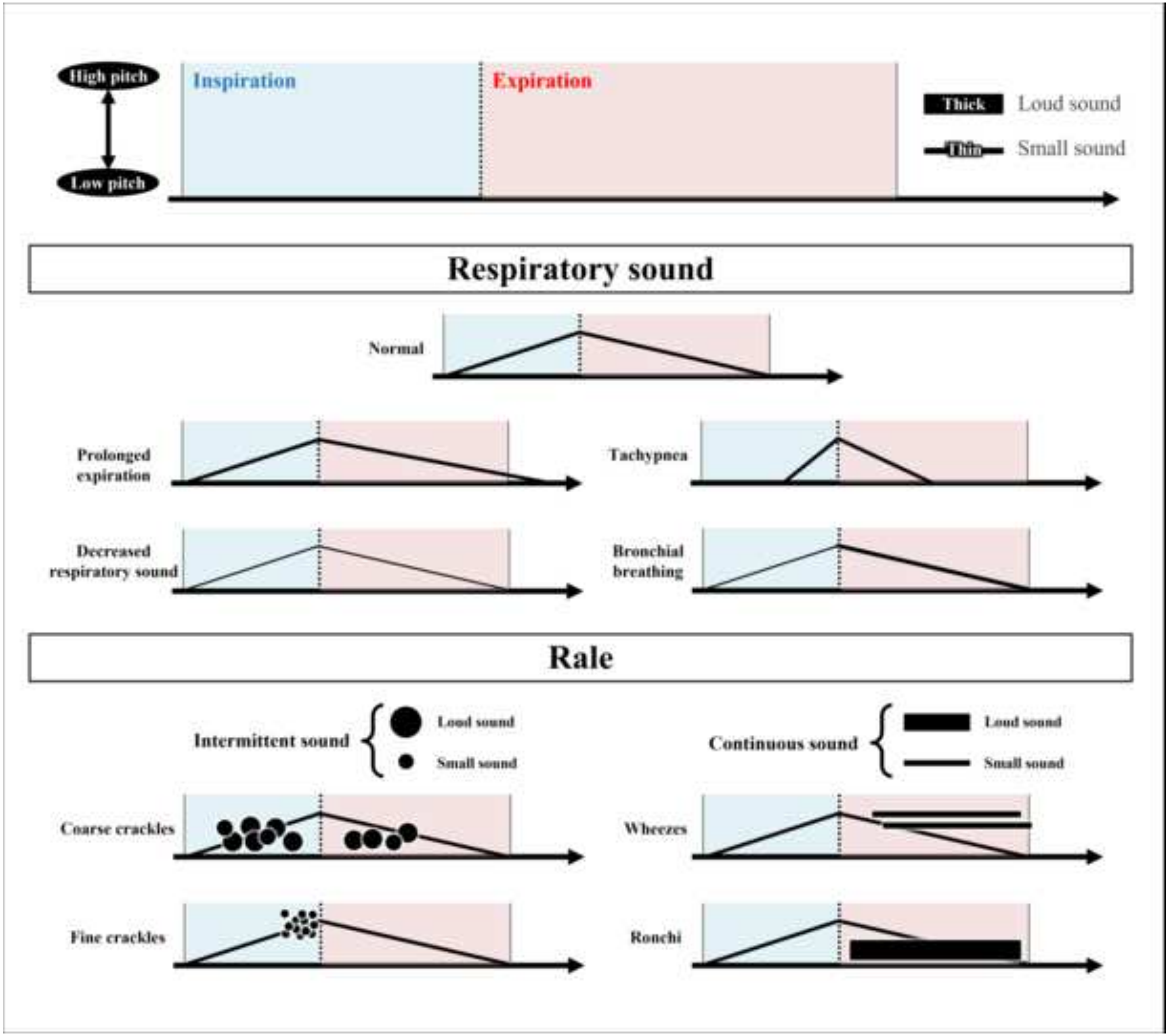

Figure 3

Click here to access/download;Figure;Figure 3.png

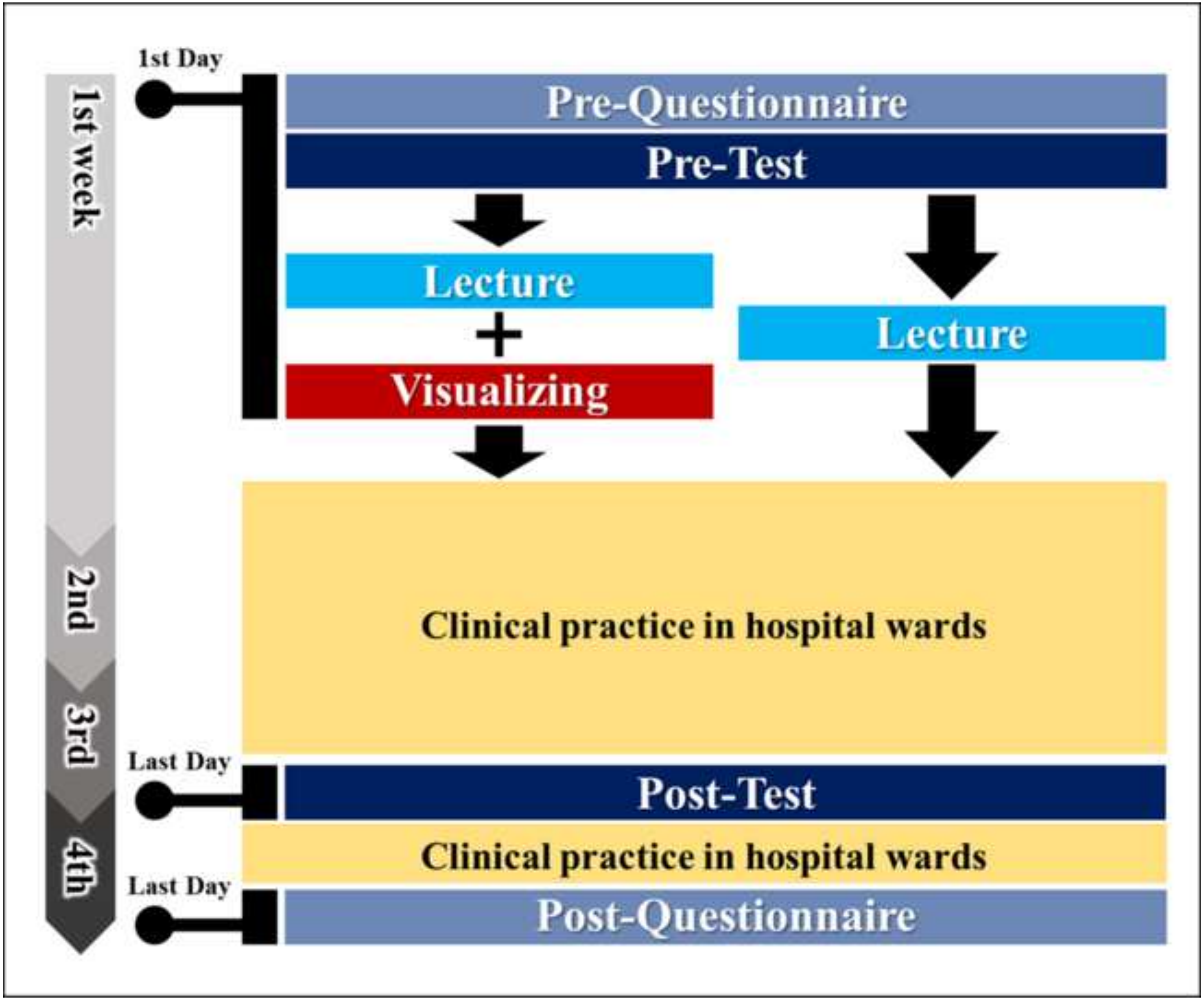

Supplementary Figure 1

Click here to access/download
Supporting Information
Supplementary figure 1.png

Supplementary Figure 2

Click here to access/download
Supporting Information
Supplementary figure 2.png

Supplementary Figure 3

Click here to access/download
Supporting Information
Supplementary figure 3.png
