# Supplementary material for: The effects of simple graphical and mental visualization of lung sounds in teaching lung auscultation during clinical clerkship: A preliminary study
Source: PLoS One. 2023 Mar 17;18(3):e0282337. doi: 10.1371/journal.pone.0282337 (PMC10022769; doi:10.1371/journal.pone.0282337)
Supplement: Attachment — Submitted filename: Response_to_Reviewers_Plos_One_0118.docx [file pone.0282337.s012.docx]

January 20, 2023

Ayse Hilal Bati, MD, PhD

Academic Editor

*PLOS ONE*

Dear Dr. Ayse

Ref. No.: PONE-D-22-25852RI

Thank you for your email dated January 5, 2023, regarding our manuscript, “**The effects of simple graphical and mental visualization of lung sounds in teaching lung auscultation during clinical clerkship: a preliminary study.**”

We thank you and the reviewers for your insightful comments and insights, based on which we have revised our manuscript. Our point-by-point responses to the reviewers’ comments are attached herewith. The changes to the manuscript are marked in red font.

We believe that our revised manuscript has suitably incorporated the reviewers’ suggestions and is significantly improved over our initial submission. We trust that it is now suitable for publication in *PLOS ONE*.

Thank you for considering our paper for publication.

Sincerely,

Hajime Kasai, MD

Department of Respirology, Graduate School of Medicine

Chiba University, 1-8-1 Inohana, Chuou-ku Chiba 260-8670, Japan

Telephone: 81-43-222-7171 Ext.71014

Fax: 81-43-226-2176

E-mail: daikasai6075@yahoo.co.jp

**Response to Reviewer #1’s comments**

**General Comment:**

Thank you for your thoughtful edits to your paper. It is improved, though I still have a few concerns that I'd like you to address in another revision:

**Response:**

We wish to express our strong appreciation for your insightful comments on our manuscript. The comments have helped us to significantly improve the manuscript. We have marked the relevant changes in red so that you can easily locate them within the manuscript.

**Comment #1:** Line 65 - change to read "education on how to perform lung.."

**Response:**

According to the comment, we have reworded the related sentence as follows:

(p4. 2nd paragraph of Introduction)

**Original:** “However, effective education for lung auscultation has not been developed.”

**Revised:** “However, effective education on how to perform lung auscultation has not been developed.”

**Comment #2:** Lines 62-82: the order of this paragraph needs to be adjusted. I would suggest moving lines 68-71 to the end of line 64, as you were previously speaking about learning from recordings. This paragraph seems to start about recordings and end with simulators; please restructure it so that it follows that pattern. Also, the sentence about on-the-job training seems inconsistent with the rest of the paragraph which is trying to describe how to actively teach this skill.

**Response:**

Thank you for this pertinent comment.

We agree with you on this point and have adjusted the order of the sentences.

We had placed the sentence about on-the-job training to emphasize the need for more effective learning in medical school, since after graduation, residents will need to learn on their own through on-the-job training. However, as you pointed out, this sentence is inconsistent with the topic of the entire paragraph. Accordingly, we have removed it.

(p4. 2nd paragraph of Introduction)

**Removed:** “Medical students are often trained in auscultation at school, and after becoming doctors, may find on-the-job training to be their main learning opportunity for lung auscultation.”

**Comment #3:** Lines 180-183 - state what Q A1 is (can paraphrase) and say it was measured on a scale of 1-5 (define 1 and 5). Then state what A2/A3 measured and state that answers were recorded on a Bohadana et al classification. Readers should only have to refer to the table if they want more info to expand on what's in the text, but what is in the text should be able to be read and understood without the table. In its current form, the text is not understandable without the table.

**Response:**

We thank the reviewer for raising this concern.

As you pointed out, it is essential for the contents of the questionnaire to be understood in the body of the text without referring to the table. Accordingly, we have added the following sentences:

(p9, 1st paragraph of Questionnaire in Methods)

**Added:** “Students were asked to rate their own lung sound auscultation ability in questions A1 and B1, whether they had any experience in listening for various lung sounds in questions A2 and B2, and their confidence in auscultating lung sounds in questions A3 and B3. In addition, after the CC, they were asked about their satisfaction level with the educational program on lung auscultation in question B4.”

**Comment #4:** Lines 180-183 - surveys are stronger when each Likert scale item is defined, but you only defined 1 and 5. This should be added as a limitation to your study.

**Response:**

Thank you for this comment.

As you pointed out, in the 5-point Likert Scale of the question regarding confidence of lung auscultation in the questionnaires for students, only value 1 and 5 were defined. Thus, the other values were self-judged by the students. More rigorous definitions for all values are needed in future study.

Accordingly, we have added the related sentence as follows.

(p19-20. Study Limitation)

**Original:** “The present study has four main limitations. First, in terms of research design, this was a single-site study with a small number of participants in an uncontrolled environment and relied partly on students’ self-assessments for data collection. Therefore, a larger number of participants based on the calculation of the required sample size and the use of a reliable questionnaire are required in the future. Second, time spent on lung auscultation education differs between the two groups with and without time spent on training to visualize lung sounds. In addition, the time taken for the self-study of lung auscultation before and during CC can be different for each student. Third, it is unclear how often students continued to visualize lung sounds in their daily practice. Fourth, in the 5-point Likert Scale of the question regarding to confidence of lung ausculation in the questionnaires for students, only value 1 and 5 were defined. Thus, the other values were self-judged by the students. More rigorous definitions for all values are needed in future study. Fifth, long-term effects have not been explored in our study. ”

**Revised:** “The present study has five main limitations. First, in terms of research design, this was a single-site study with a small number of participants in an uncontrolled environment and relied partly on students’ self-assessments for data collection. Therefore, a larger number of participants based on the calculation of the required sample size and the use of a reliable questionnaire are required in the future. Second, the time spent on lung auscultation education differs between the two groups with and without time spent on training to visualize lung sounds. This difference in time may have affected students’ confidence in auscultation as a confirmation bias. In addition, the time taken for the self-study of lung auscultation before and during CC can be different for each student. Third, it is unclear how often students continued to visualize lung sounds in their daily practice. Fourth, in the questionnaires for the students, only values 1 and 5 were defined in the 5-point Likert Scale of the question regarding confidence in lung auscultation. The other values were self-judged by the students. Thus, more rigorous definitions for all values are needed in future studies. Fifth, long-term effects have not been explored in our study.”

**Comment #5:** Table 1 - I would suggest moving this survey to be an appendix rather than a table.

**Response:**

We have changed Table 1 to Supplementary Table 1 and moved it to the Appendix.

We have also corrected some unforeseen description in questions A3 and B3, as well as a misstatement of the 5-point Likert scale value in responses for question B4 as follows.

In addition, we have renumbered other tables accordingly.

**Revised:**

**Supplementary table 1. Questionnaire items before and after the training on lung auscultation.**

| **Before the CC in Respiratory Medicine** | **Responses** |
| --- | --- |
| (A1) How is your current lung sound auscultation ability? | Five-point Likert scale  1 (Not confident [cannot listen at all]) to 5 (Confident [able to listen and distinguish between lung sounds and rales]) |
| (A2) Have you ever heard each lung sound from a patient?  Decreased respiratory sounds, bronchial breathing, prolonged expiration, coarse crackles, fine crackles, wheezes, rhonchi, squawk, and pleural friction rub | Yes or No |
| (A3) How confident are you in auscultating each lung sound?  Same items as in question A2. | The response options are same as in question A1. |
| **After the CC in Respiratory Medicine** |  |
| (B1) How is your current lung sound auscultation ability? | The response options are same as in question A1. |
| (B2) Have you ever heard each lung sound from a patient?  Same items as in question A2. | The response options are same as in question A2. |
| (B3) How confident are you in auscultating each lung sound?  Same items as in question A2. | The response options are same as in question A1. |
| (B4) What is your level of satisfaction with the education program on lung auscultation? | Five-point Likert scale  1 (extremely poor) to 5 (extremely good) |

**Comment #6:** Line 223 - please include the results of the normality testing for each type of data tested (e.g. test scores for lung auscultation; and did you also test the questionnaire answers or did you automatically assume that those were non-parametric because they are ordinal data? Except that you reported mean and SD for the questionnaire answers, which suggests you treated them as parametric data. If you treated ordinal data as non-parametric, then you should have reported median instead of means.)

**Response:**

Thank you for this comment.

As shown in the previous response, only scores of lung auscultation tests in control group were normally distributed (Table A). Therefore, we have added Table A excluding rows for age and Results of CC in Respiratory Medicine as Supplementary Table 2.

**Table A.** P-value in the test of normality of each parameter (n=65).

|  | Visualization group (n = 35) | Control group  (n = 30) |
| --- | --- | --- |
| Age | ＜0.001 | ＜0.001 |
| Results of CC in Respiratory Medicine | 0.780 | 0.012 |
| Satisfaction | ＜0.001 | ＜0.001 |
| Confidence |  |  |
| Pre-questionnaire | ＜0.001 | 0.010 |
| Post-questionnaire | ＜0.001 | ＜0.001 |
| Score of lung auscultation |  |  |
| Pre-test | 0.008 | 0.610 |
| Post-test | 0.049 | 0.747 |

We treated all parameters as a nonparametric value to unify the statistical testing methods. Hence, we have presented their medians and interquartile range (IQR) in Table B.

We have also changed the values in Table 2 (Original: Table 3), as shown in Table B.

**Table B.** **Satisfaction level with our education and changes in the confidence level pertaining to lung auscultation and the score of lung auscultation before and after education (*n* = 65).**

|  | Visualization group (n = 35) | Control group  (n = 30) | p-value |
| --- | --- | --- | --- |
| Satisfaction, median (IQR) | 5 (1) | 4 (1) | 0.150 |
| Confidence in lung auscultation |  |  |  |
| Pre-questionnaire, median (IQR) | 1 (1) | 2 (1) | 0.128 |
| Post-questionnaire, median (IQR) | 3 (1) | 3 (1) | **0.028** |
| Mean difference, median (IQR) | 2 (1) | 1 (1) | **0.005** |
| Adjusted mean difference, least mean square (SE) | 1.7 (0.1) | 1.3 (0.1) | **0.020*** |
| Score of lung auscultation |  |  |  |
| Pre-test, median (IQR) | 11 (2) | 11 (5) | 0.827 |
| Post-test, median (IQR) | 15 (4) | 14 (4) | 0.290 |
| Mean difference, median (IQR) | 4 (4) | 3 (1) | 0.623 |
| Adjusted mean difference, least mean square (SE) | 3.7 (0.5) | 3.1 (0.6) | 0.424* |

* Analysis of covariance (ANCOVA) was performed, and pre-score was adjusted.

Interquartile range, IQR; standard error, SE.

Accordingly, we have changed the related sentences as follows:

(p2. Result in Abstract)

**Original:** “Confidence in auscultation of lung sounds significantly increased in both groups (five-point Likert scale, visualization group: pre 1.5±0.1 to post 3.2±0.1, p<0.001; control group: 1.7±0.1 to 2.9±0.1, p<0.001), and was significantly higher in the visualization than in the control group. Moreover, test scores increased in both groups (visualization group: pre-test 11.4±0.5 to post-test 15.1±0.6, p<0.001; control group: 11.1±0.5 to 14.3±0.6, p<0.001).”

**Revised:** “Confidence in auscultation of lung sounds significantly increased in both groups (five-point Likert scale, visualization group: pre-questionnaire median 1 [Interquartile range 1] to post-questionnaire 3 [1], p<0.001; control group: 2 [1] to 3 [1], p<0.001) and was significantly higher in the visualization than in the control group. Test scores increased in both groups (visualization group: pre-test 11 [2] to post-test 15 [4], p<0.001; control group: 11 [5] to 14 [4], p<0.001).”

(p11. Statistical analysis in Methods section)

**Original:** “All results are expressed in terms of mean ± standard deviation (SD), unless otherwise indicated.”

**Revised:** “All results are expressed in terms of the median and interquartile range (IQR) unless otherwise indicated.”

(p12. 1st paragraph of Result section)

**Original:** “The visualization group included 35 students (age 23.2±1.6 years; male/female 27/8), and the control group included 30 students (22.6±0.8 years; male/female 24/6) (Fig 1). There were no significant differences in the age and sex ratio (*p*=0.123 and *p*=0.780, respectively). In addition, there was no significant difference in the results of CC in Respiratory Medicine, which did not include the results of the lung auscultation test between the two groups (maximum score: 25, 76.7±3.9 vs. 77.1±5.1, *p*=0.802).”

**Revised:** “The visualization group included 35 students (age median 23 [IQR 2]; male/female 27/8), and the control group included 30 students (22 [IQR 1] years; male/female 24/6) (Fig 1). There were no significant differences in the age and sex ratio (*p*=0.123 and *p*=0.780, respectively). In addition, there was no significant difference in the results of CC in Respiratory Medicine, which did not include the results of the lung auscultation test between the two groups (maximum score: 100, 76.4 [5.8] vs. 76.5 [5.4], *p*=0.802).”

(p12. 1st paragraph of Questionnaire in Result section)

**Original:** “Based on an analysis of their responses, students’ satisfaction level with our education was high in both groups, but there was no significant difference (visualization group: 4.5±0.7 vs. control group: 4.2±0.9, p = 0.150).”

**Revised:** “Based on an analysis of their responses, students’ satisfaction level with our education was high in both groups, but there was no significant difference between groups (visualization group: median 5 [IQR 1] vs. control group: 4 [1], *p* = 0.150).”

(p13. 2nd paragraph of Questionnaire in Result section)

**Original:** “Confidence in lung auscultation (A1 and B1: “How is your current lung sound auscultation ability?”) significantly increased in both groups (five-point Likert scale; visualization group, pre-test: 1.5±0.6 to post-test: 3.3±0.7, p < 0.001; control group, 1.7±0.6 to 2.9±0.8, p < 0.001). Confidence in lung auscultation after CC was found to be significantly higher in the visualization group than in the control group (p = 0.028; adjusted mean difference between pre- and post-test, 1.7±0.1 vs. 1.3±0.1, p = 0.020).”

**Revised:** “Confidence in lung auscultation (A1 and B1: “How is your current lung sound auscultation ability?”) significantly increased in both groups (five-point Likert scale; visualization group, pre-questionnaire: 1 [1] to post-questionnaire: 3 [1], *p* < 0.001; control group, 2 [1] to 3 [1], *p* < 0.001). Confidence in lung auscultation after CC was found to be significantly higher in the visualization group than in the control group (*p* = 0.028; adjusted mean difference between pre- and post-questionnaire, 1.7 [standard error 0.1] vs. 1.3 [0.1], *p* = 0.020).”

(p13. 1st paragraph of Results of tests of lung auscultation in Result section)

**Original:** “The test score increased in both groups (visualization group, pre- 11.4±2.5 to post-test 15.1±3.5, p < 0.001; control group, 11.1±3.3 to 14.3±3.2, p < 0.001) as shown in Table 3. There was no difference between the scores of the pre-and post-tests for both groups (pre-test, p = 0.827; post-test, p = 0.290). In addition, there was no change in scores between the two groups (adjusted mean difference between pre-and post-tests, 3.7±0.5 vs. 3.1±0.6, p = 0.424).”

**Revised:** “The test score increased in both groups (visualization group, pre-test 11 [2] to post-test 15 [4], *p* < 0.001; control group, 11 [5] to 14 [4], *p* < 0.001) as shown in Table 2. There was no difference between the scores of the pre-and post-tests for both groups (pre-test, *p* = 0.827; post-test, *p* = 0.290). In addition, there was no change in scores between the two groups (adjusted mean difference between pre-and post-tests, 3.7 [standard error 0.5] vs. 3.1 [0.6], *p* = 0.424).”

**Comment #7:** Line 225 - how and why did you adjust the values?

**Response:**

Thank you for this query.

In this study, confidence in and scores on lung auscultation in the two groups before and after the lecture were compared using analysis of covariance (ANCOVA), by adjusting the values of the pre-questionnaire and pre-test results for each group. The change in the values of the pre-questionnaire and pre-test results was adjusted as an outcome for the comparison. Moreover, these adjustments were performed to avoid any misalignment of the values of each parameter when comparing the groups.

Accordingly, we have revised the related sentences as follows:

(p11-12, Statistical analysis in Methods)

**Original:** “Confidence in and scores on lung auscultation in the two groups before and after our lecture were compared using analysis of covariance (ANCOVA), by adjusting the values of the pre-questionnaire and pre-test results for each group.”

**Revised:** “Confidence in and scores on lung auscultation in the two groups before and after our lecture were compared using analysis of covariance (ANCOVA), by adjusting the values of the pre-questionnaire and pre-test results for each group. Change of confidence in and scores on lung auscultation in the two groups before and after our lecture were compared using multivariate linear regression. In the multivariate analysis, the values of the pre-questionnaire and pre-test results were adjusted.”

**Comment #8:** Lines 239-241 - normality testing results should go in statistical methods not results.

**Response:**

Thank you for this important suggestion.

Accordingly, we have moved the sentences as follows:

**(p11. Statistical analysis in Methods)**

**Original:** “All results are expressed in terms of mean ± standard deviation (SD), unless otherwise indicated. The Wilcoxon signed-rank test was used to compare the questionnaire answers about participants’ confidence in lung auscultation and the test scores of lung auscultation before and after the training. Normality was evaluated using the Shapiro-Wilk test. Confidence in and scores on lung auscultation in the two groups before and after our lecture were compared using analysis of covariance (ANCOVA), by adjusting the values of the pre-questionnaire and pre-test results for each group. A p-value < 0.05 was considered statistically significant. All statistical analyses were performed using JMP 15.0 software (Cary, North Carolina, USA) and SAS software version 9.4 (SAS Institute, Cary, USA).”

**Revised:** “All results are expressed in terms of the median and interquartile range (IQR) unless otherwise indicated. Normality was evaluated by performing the Shapiro–Wilk test. In the data collected in this study, the pre- and post-tests for the control group were normally distributed, while the other parameters were non-normally distributed (Supplementary table 2). The Wilcoxon signed-rank test was used to compare the questionnaire answers about participants’ confidence in lung auscultation and the test scores of lung auscultation before and after the training. Confidence in and scores on lung auscultation in the two groups before and after our lecture were compared using analysis of covariance (ANCOVA), by adjusting the values of the pre-questionnaire and pre-test results for each group. Change of confidence in and scores on lung auscultation in the two groups before and after our lecture were compared using multivariate linear regression. In the multivariate analysis, the values of the pre-questionnaire and pre-test results were adjusted. A *p-*value < 0.05 was considered statistically significant. All statistical analyses were performed using JMP 15.0 software (Cary, North Carolina, USA) and SAS software version 9.4 (SAS Institute, Cary, USA).”

**Comment #9:** Line 244 - add "between groups" after significant difference.

**Response:**

According to your suggestion, we have reworded the relevant sentence as follows:

(p12. 1st paragraph of Questionnaire in Result section)

**Original:** “Based on an analysis of their responses, students’ satisfaction level with our education was high in both groups, but there was no significant difference (visualization group: 4.5±0.7 vs. control group: 4.2±0.9, p = 0.150).”

**Revised:** “Based on an analysis of their responses, students’ satisfaction level with our education was high in both groups, but there was no significant difference between groups (visualization group: median 5 [IQR 1] vs. control group: 4 [1], *p* = 0.150).”

**Comment #10:** Lines 296-299 - or they are more confident because they have undergone more training? Students who have completed more training feel more confident because of confirmation bias (if they spend more time on something, their mind wants to confirm that was valuable time spent, so they increase in confidence to perform). You need to include this (more confident due to more instructional time) as a possibility too.

**Response:**

Thank you for this comment.

As you noted, the time for lecture on lung auscultation was longer in the visualization group. The additional time added to the common part of the lecture on lung sound auscultation for the two groups was 30 minutes, which was a relatively short time for the duration of the 4-week CC. Moreover, we did not inform the participants that the lecture time was different between the visualization and control groups.

However, the possibility of the influence of confirmation bias on the improvement of confidence cannot be ruled out.

Accordingly, we have added it as the third limitation under Study Limitation.

(p19, Study limitation)

**Original:** “Second, time spent on lung auscultation education differs between the two groups with and without time spent on training to visualize lung sounds. In addition, the time taken for the self-study of lung auscultation before and during CC can be different for each student.”

**Revised:** “Second, the time spent on lung auscultation education differs between the two groups with and without time spent on training to visualize lung sounds. This difference in time may have affected students’ confidence in auscultation as a confirmation bias. In addition, the time taken for the self-study of lung auscultation before and during CC can be different for each student. ”

**Comment #11:** Line 356 - change evaluate to evaluated.

**Response:**

According to the comment, we have changed the related sentence as follows:

(p18. 7th paragraph of Discussion)

**Original:** “In this study, students in the visualization group drew lung sound diagrams only as an aid during the post-test. In the future, students will be provided with multiple opportunities to draw lung sounds and their lung sound diagrams will be evaluate.”

**Revised:** “In this study, students in the visualization group drew lung sound diagrams only as an aid during the post-test. In the future, students will be provided with multiple opportunities to draw lung sounds and their lung sound diagrams will be evaluated.”

**Comment #12:** Lines 371-372 - you can't say that it may improve skills (your p value was not significant) but can only say it may improve or does improve confidence.

**Response:**

Thank you for this suggestion.

As you pointed out, our educational program could only improve students’ confidence in lung auscultation. Accordingly, we have revised the related sentence as follows:

(p19. Conclusion)

**Original:** “The visualization of lung sounds may improve medical students’ lung auscultation skills and their confidence in auscultation.”

**Revised:** “The visualization of lung sounds may improve medical students’ confidence in lung auscultation.”
